# Asymmetric seasonal daytime and nighttime warming and its effects on vegetation in the Loess Plateau

**DOI:** 10.1371/journal.pone.0218480

**Published:** 2019-06-24

**Authors:** Liqun Ma, Fen Qin, Hao Wang, Yaochen Qin, Haoming Xia

**Affiliations:** 1 College of Environment and Planning, Key Laboratory of Geospatial Technology for Middle and Lower Yellow River Regions Ministry of Education, Henan Collaborative Innovation Center of Urban-Rural Coordinated Development, Henan University, Kaifeng, Henan Province, China; 2 School of Civil Engineering and Architecture, Henan University, Kaifeng, Henan Province, China; Universite du Quebec a Chicoutimi, CANADA

## Abstract

Over the period 1982–2015, temperatures have exhibited an asymmetric warming pattern diurnally, as well as seasonally across the Loess Plateau. However, very limited research has studied the implications and effects of such seasonally heterogeneous warming across the Loess Plateau. In this study, we also analyzed the time series trends and seasonal spatial patterns of the maximum (*T*_*max*_) and minimum (*T*_*min*_) temperatures and evaluated how different vegetation responded to daytime and nighttime warming in the Loess Plateau from 1982 to 2015 based on the NDVI and meteorological parameters (precipitation or temperature). We found that *T*_*max*_ and *T*_*min*_ significantly increased throughout the years except for *T*_*max*_ in autumn, and the diurnal asymmetric warming showed some striking seasonal differences. For example, the increasing rates of *T*_*min*_ in spring, summer, autumn, and winter were 0.75, 1.20, 1.88, and 1.10 times larger than that of *T*_*max*_, respectively. NDVI showed significantly positive correlation with *T*_*max*_ and *T*_*min*_ in spring and winter, while NDVI presented significantly positive correlation with *T*_*min*_ in summer and *T*_*max*_ in autumn across entire Loess Plateau. Furthermore, we also discovered diverse seasonal responses in terms of vegetation types to daytime and nighttime warming. For instance, Spring NDVI showed significantly positive partial correlations with *T*_*max*_ and *T*_*min*_. In summer, grasslands and wetlands merely displayed significantly positive partial correlations with *T*_*min*_. Cultivated land presented significantly positive partial correlation between the NDVI and *T*_*max*_ (*T*_*min*_) in autumn. In winter, cultivated land, forest, and grassland exhibited significantly positive partial correlation with *T*_*max*_ and *T*_*min*_, while only wetland showed a significantly positive partial correlation with *T*_*max*_. Our results demonstrated responses of vegetation to climate extremes and enhance a better understanding of the seasonally different responses of vegetation under global climate change at different scale.

## 1. Introduction

Vegetation is an important part of the global terrestrial ecosystem, playing as a pivotal medium in the flows of both matter and energy across the atmosphere, hydrosphere, biosphere, and pedosphere [1]. Vegetation growth is closely related to climatic factors and hydrothermal processes [2–3]. Temperature and precipitation are two dominant climatic factors that influence vegetation growth and production [2]. The relationship between vegetation growth and climatic factors typically varies with different vegetation types and different spatiotemporal scales [2–3]. However, most of the previous studies have focused on revealing the impact of the average state of climatic factors on vegetation growth [4–5]. The effects of seasonal extreme temperature change on vegetation are poorly understood.

Recently, a growing number of studies have shown that the global climate has shown asymmetric annual and seasonal daytime and nighttime warming [3,6–7]. The trend of the nighttime minimum temperature (*T*_*min*_) was faster than that of the daytime maximum temperature (*T*_*max*_) [6], which is known as asymmetric warming [3]. The increasing rate of minimum temperature was about twice of the maximum temperature over global land areas from 1950 [3]. In summer, the increasing rate of temperatures in the high latitudes of the northern hemisphere is faster than that in spring and autumn, and the seasonal temperature difference shows a downward trend [8]. Accordingly, the changing characteristics of global temperature rise and seasonal daytime and nighttime asymmetric warming have been proved to affect plant photosynthesis activity and yield differently [5,6–7]. The differences in the physiological and ecological characteristics of vegetation types lead to different responses to asymmetric warming [3]. Therefore, it is necessary to study the effects of seasonal daytime and nighttime asymmetric warming on vegetation dynamic changes.

Until now, previous studies have investigated the responses of the modes of the ecosystems to annual and seasonal daytime and nighttime asymmetric warming through ground control experiments, model simulations, and remote sensing data analysis [3, 6–7]. Dynamic responses of different vegetation to asymmetric warming are intriguing, but their explanations are still uncertain. For instance, some studies have showed that *T*_*max*_ is beneficial to most vegetation growth in wetter and colder regions but negatively correlates with normalized difference vegetation index (NDVI) in arid and semi-arid regions, while the increasing *T*_*min*_ inhibits the growth of vegetation [3]. On the contrary, Xia et al. [7] have reported that *T*_*min*_ increases positively correlate with vegetation in the high-cold steppe and meadow steppe zones, while *T*_*max*_ increases are significantly positively correlated with GIMMS3g NDVI in wetter and colder regions of the Tibetan Plateau. Using MODIS, SPOT NDVIs, and MODIS EVI, Shen et al. [9] found that maximum temperature increases in summer in the Tibetan Plateau are conducive to the growth of vegetation, but the temperature increases at night restrain vegetation growth.

The response of vegetation growth to asymmetric warming also shows a seasonal variation [7]. For instance, Piao et al. [10] found that net carbon uptake of northern ecosystems is being decreased in response to autumnal warming. Using GIMMS3g NDVI, Xu et al. [11] reported that the increasing of temperature in spring enhances vegetation growth in Central Eurasia after 2002, while in summer vegetation growth is mainly driven by precipitation. Many other studies have further shown that seasonal daytime and nighttime warming has different effects on vegetation [12–13]. Wu et al. [12] found that *T*_*max*_ is significantly correlated with the GIMMS3g NDVI in vast boreal forests of the Northern Hemisphere in spring, summer and autumn, but *T*_*max*_ is negatively related with the vegetation growth in temperate dry ecosystems in summer. The *T*_*min*_ is positively correlated with the NDVI in spring and summer, but *T*_*min*_ is negatively correlated with the NDVI in autumn, using NDVI data from 1982 to 2011 from the AVHRR. Using NDVI data from 1982 to 2011 obtained from the AVHRR, Tan et al. [13] showed that the *T*_*max*_ increases prevent the growth of vegetation in arid regions in summer, but is conducive to the growth of vegetation in cold regions in spring. The increasing of *T*_*min*_ is beneficial to vegetation growth in spring and summer but restrains most vegetation growth in autumn [13]. In summary, different intensity of responses to diurnal asymmetry warming has showed complex correlations due to vegetation types, regions, and seasons.

The Loess Plateau is located in the transition zone of semi-humid and semi-arid climates [14], and it is sensitive to climate change. This area has a fragile ecological system, containing a key soil and water conservation area in the middle and upper reaches of the Yellow River [15]. The different hydrothermal distributions in different seasons lead to diverse responses of vegetation across regions and seasons. At the same time, the special natural environment of the Loess Plateau is also the place that can be used to verify the relationships between vegetation and hydrothermal climate change at local scale.

The satellite-based NDVI, which has been used successfully in long-term monitoring of vegetation dynamics at regional and global scales, has proven to be a robust indicator of vegetation growth [16]. When NDVI, with different spatial and temporal resolutions from different platforms and sensors, is used to monitor the vegetation dynamics, the results may have some differences. Therefore, it is very important to select suitable NDVI for the given research purpose. The widely used NDVI including the AVHRR, MODIS, and SPOT VEGETATION has been proven to be a robust indicator of vegetation changes at regional and global scales for long-term temporal examinations [2–3]. In order to detect long-term vegetation dynamics, the third-generation Normalized Difference Vegetation Index (NDVI3g) dataset, which released by the NASA’s Global Inventory Modeling and Mapping Studies (GIMMS) group, is selected to monitor vegetation change. At the same time, NDVI3g data has been normalized to account for sensor calibration loss, orbital drift, and atmospheric effects and has been proved its good ability for the long-term monitoring of vegetation changes.

Using the GIMMS3g NDVI and meteorological (temperature and precipitation) data during 1982–2015, we use the Mann-Kendall non-parametric hypothesis tests and the second-order partial correlation analysis method to determine the time series trends and spatial patterns of *T*_*max*_ and *T*_*min*_ in different seasons on the Loess Plateau as well as the responses of different vegetation types to seasonal daytime and nighttime asymmetric warming. The results can provide an evaluation of changes in vegetation and ecological health in the Loess Plateau in response to climate change.

## 2. Materials and methods

### 2.1. Study area

**Fig 1:**
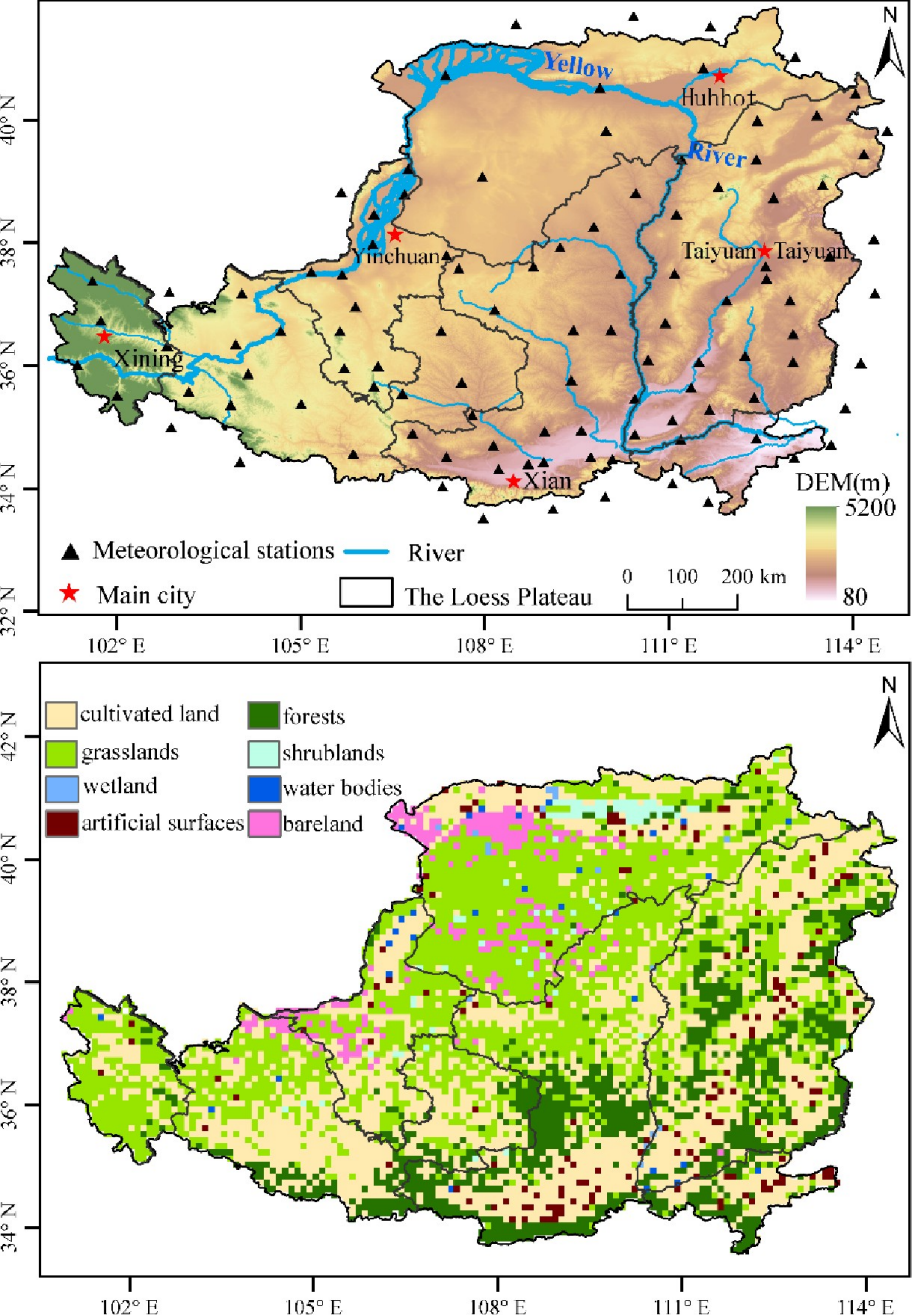
The altitude, meteorological stations, and vegetation types across the Loess Plateau.

The Loess Plateau lies in the upper and middle reaches of the Yellow River from 100°54′E to114°33′E and from 33°43′N to 41°16′N, covering an area of approximately 624,000 km^2^ (Fig 1). From south to north, there are two heat zones: a warm temperate zone and medium temperature zone. From east to west, the Loess Plateau includes semi-humid, semi-arid, and arid areas. It has a typical continental monsoon climate. According to the meteorological stations in the area, the average annual temperature is between 9°C and 12°C during 1982–2015 [17]. The vegetation dynamic changes and the temperature differences in the east and west are large. The Loess Plateau is characterized by a rainy summer and autumn, with less rain in the winter and spring. Precipitation is mostly restricted from July to September, and the average annual precipitation varied from 100 to 800 mm from northwest to southeast in 1982–2015 (Fig 1).

### 2.2. Data and processing

#### 2.2.1. Meteorological data and preprocessing

We obtained the meteorological data (temperature and precipitation) from the China Meteorological Data Sharing Service System (http://cdc.cma.gov.cn/home.do) [18]. Data quality control is a prerequisite for index calculations, and we performed the data quality processes using the computer program RClimDex Software Version 1.1, which is developed and maintained by researchers at the Climate Research Branch of the Meteorological Service of Canada [19]. Using the detailed principles described by Wang [20], we conducted a homogeneity test with the RHtest V4 software to identify possible change points or structural changes in the data series. The only criterion for inclusion of a meteorological station was that its recording period must cover at least 85% of the total 34-year period. After data quality control and a homogeneity assessment, 34-year periodical records at 106 stations (Fig 1) were selected (from 1 January 1982 to 31 December 2015).

According to previous research, the spatial interpolation for temperature based on a DEM (digital elevation model) provided by the NASA Shuttle Radar Topographic Mission (SRTM) (http://www.glcf.umd.edu) can effectively simulate the spatial distribution of temperature [21]. This study used the DEM as a co-variable and employed thin plate smoothing splines to interpolate the temperature and precipitation data. The final interpolation outputs were 8 km×8 km grids of data.

According to a typical practice [22], one year was divided into four seasons: spring (March-May), summer (June-August), autumn (September-November), and winter (December-February).The *T*_*max*_ and *T*_*min*_ in different seasons were calculated based on the average of the daily maximum and minimum temperature in the same season over years, respectively. The precipitation was defined as the accumulated precipitation for the corresponding season.

#### 2.2.2. GIMMS-NDVI3g and preprocessing

The GIMMS-NDVI3g dataset is published by Global Inventory Modeling and Mapping Studies, which is based on a synthesis method using the maximum value with a time interval of 15 days, a spatial resolution of 8 km×8 km, and a time series covering the period 1982–2015 [23]. This dataset has been calibrated for radiation and atmospheric correction and coordinate transformation; images of the daily satellite tracks are available with geometric correction, cloud-removal, and removal of bad lines for NDVI calculation and data generation [23]. To further eliminate noise and clouds [23], we used the maximum synthesis method to synthesize monthly data by IDL. The pixels with an average season NDVI value larger than 0.1 were only considered, so that the influence of soil variation on the spectral signal in bare and sparsely vegetated zones could be minimized [24].

#### 2.2.3. Vegetation data and preprocessing

The GlobeLand30 data are freely available and included eight types of land cover including forests, cultivated land, grasslands, shrublands, bareland, wetland, water bodies and artificial surfaces across Loess Plateau [25]. We selected forests, cultivated land, grasslands, shrublands, wetland in this study. The vegetation data were resampled to match the spatial resolution of GIMMS NDVI3g data using a majority filter method.

### 2.3. Data analysis

#### 2.3.1. Mann-Kendall

The Mann-Kendall non-parametric hypothesis test method has advantage that samples do not have to conform to a certain distribution, and abnormal values little impact on the test results [26]. Therefore, we adopt Mann-Kendall to detect the change trend of *T*_*max*_, *T*_*min*_, and NDVI. The slope of *T*_*max*_, *T*_*min*_, and NDVI were calculated using linear regression at the regional and pixel scales, respectively.

#### 2.3.2 Partial correlation analysis

Partial correlation yields the correlation between two variables, controlling for a third or more variables [27]. The second-order partial correlation analysis was used to distinguish the influence of interannual changes in *T*_*max*_ and *T*_*min*_ on vegetation NDVI among different seasons. The partial correlation coefficient between NDVI and *T*_*max*_ in each season was calculated by controlling *T*_*min*_ and precipitation, and likewise, the partial correlation coefficient between the NDVI and *T*_*min*_ in each season was calculated by controlling *T*_*max*_ and precipitation.

## 3. Results and analysis

### 3.1. Spatiotemporal patterns of asymmetric warming and NDVI

#### 3.1.1. The regional analysis

*T*_*max*_ increased significantly in spring, summer, and winter (*p<*0.05) and non-significantly in autumn (*p*>0.05) during the 1982–2015 period (Table 1). *T*_*max*_ had the highest and most significant temperature increases in spring (0.74°C/10 years) and summer (0.42°C/10 years), with a low speed rise in autumn. *T*_*min*_ also showed a significant increasing trend in all seasons (*p*<0.05) with high speed rises in spring (0.55°C/10 years) and summer (0.51°C/10 years), and the lowest speed rise in winter (0.47°C/10 years). The rate of increase of *T*_*max*_ in spring was 1.3 times faster than that of *T*_*min*_, whereas *T*_*min*_ increased 1.2 times, 1.9 times, and 1.1 times more than *T*_*max*_ in summer, autumn, and winter, respectively.

**Table 1:** Statistics of trends in seasonal *T*_*max*_ /*T*_*min*_ and NDVI from 1982 to 2015.

	*T*_*max*_	*T*_*min*_	NDVI
Spring	0.074**	0.055**	0.001**
Summer	0.042**	0.051**	0.002**
Autumn	0.026^NS^	0.499**	0.002**
Winter	0.043**	0.047**	0.0004^NS^

NS (non-significant): *p >* 0.05,

** *p* < 0.01.

The seasonal NDVI showed a significant (*p*< 0.01) increasing trend during the study period except for winter.

#### 3.1.2. The pixel scale analysis

The trends of temperature always show spatial heterogeneity due to the variation characteristics of land surface [28]. The spatial of average temperature cannot effectively describe the variation characteristics in different regions [29].

**Fig 2:**
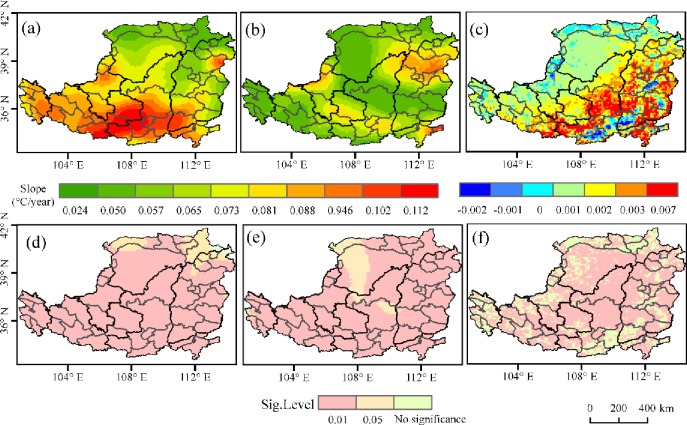
Trends of *T*_*max*_ /*T*_*min*_ and NDVI in spring over the Loess Plateau from 1982 to 2015. (a) the slope of *T*_*max;*_ (b) the slope of *T*_*min*_; (c) the slope of NDVI; (d) the significance level of *T*_*max*_ trends; (e) the significance level of *T*_*min*_ trends; and (f) the significance level of NDVI trends.

*T*_*max*_ of the Loess Plateau in spring showed an increasing trend from 1982 to 2015 and 99.1% of the area showed a significant increasing trend (*p<*0.05), among which 90.9% showed the highest and most significant increasing trend (*p*<0.01) (Fig 2A). The areas with non-significant increasing trend of *T*_*max*_ in spring (*p*>0.05) were mainly distributed in north of this area. The area of *T*_*min*_ showing a significant increasing trend was 99.7% (*p*<0.05), among which 90.3% showing a very significant increasing trend (*p*<0.01). The areas showing non-significant increasing trends of *T*_*min*_ in spring (*p*>0.05) were mainly distributed in northwest of the area (Fig 2B).

The area that has an increasing trend of NDVI in spring during the study period was 88.9%. 78.7% of the area has a significant increasing trend(*p*<0.05). The areas showing significant increasing trends of NDVI in spring (*p*>0.05) were mainly distributed in middle of this area (Fig 2C).

**Fig 3:**
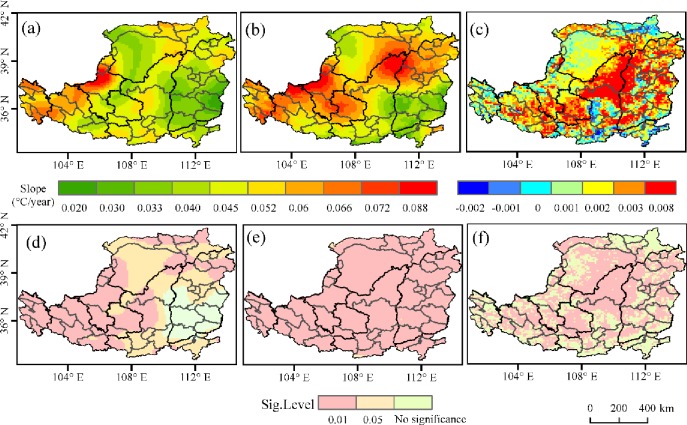
Trends of *T*_*max*_ /*T*_*min*_ and NDVI in summer over the Loess Plateau from 1982 to 2015. (a) the slope of *T*_*max;*_ (b) the slope of *T*_*min*_; (c) the slope of NDVI; (d) the significance level of *T*_*max*_ trends; (e) the significance level of *T*_*min*_ trends; and (f) the significance level of NDVI trends.

*T*_*max*_ in summer had a significant increasing trend is 85.5% (*p<*0.05) of the area, among which 47.8% had a highly significant increasing trend (*p*<0.01) (Fig 3). Areas with non-significant increasing *T*_*max*_ trends in summer (*p*>0.05) were mainly distributed in east of the area (Fig 3A). *T*_*min*_ had a significant increasing trend in summer over the Loess Plateau (*p<*0.05) (Fig 3B).

The area that has an increasing trend of NDVI in summer from1982 to 2015 was 87.5%. 69.1% of the area has a significant increasing trend (*p*<0.05). The areas showing significant increasing trends of NDVI (*p*>0.05) were mainly distributed in middle of the area (Fig 3C).

**Fig 4:**
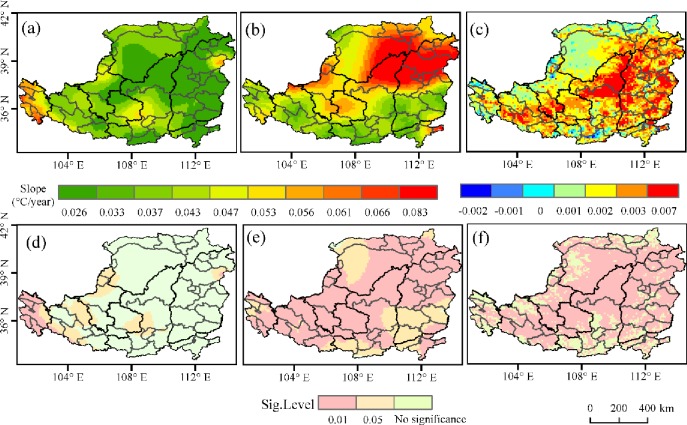
Trends of *T*_*max*_ /*T*_*min*_ and NDVI in autumn over the Loess Plateau from 1982 to 2015. (a) the slope of *T*_*max*_; (b) the slope of *T*_*min*_; (c) the slope of NDVI; (d) the significance level of *T*_*max*_ trends; (e) the significance level of *T*_*min*_ trends; and (f) the significance level of NDVI trends.

In autumn, *T*_*max*_ in most areas showed an increasing trend, among which of 18.8% was a significant trend (*p <* 0.05), mainly in west of the area. Only 3% of the regions showed a decreasing trend (*p>*0.05), mainly in the northeast of the area (Fig 4A). *T*_*min*_ showed a significant increasing trend was 99.3% of the region, and the fastest increasing trends occurred in northeast of the area(*p*<0.05) (Fig 4B).

The area that has an increasing trend of NDVI in autumn from1982 to 2015 was 95.3%. 81.2% of the area has a significant increasing trend (*p*<0.05). The areas showing non-significant increasing trends of NDVI (*p*>0.05) were mainly distributed in south and northwest of the area (Fig 4C).

**Fig 5:**
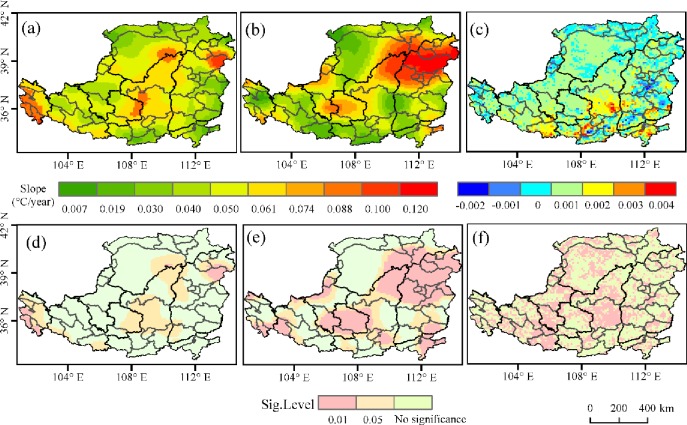
Trends of *T*_*max*_ /*T*_*min*_ and NDVI in winter over the Loess Plateau from 1982 to 2015. (a) the slope of *T*_*max;*_ (b) the slope of *T*_*min*_; (c) the slope of NDVI; (d) the significance level of *T*_*max*_ trends; (e) the significance level of *T*_*min*_ trends; and (f) the significance level of NDVI trends.

In winter, *T*_*max*_ showed a significant increasing trend in 26.4% (*p<*0.05) of the area, mainly in middle and west of area (Fig 5A). *T*_*min*_ showed a downward trend in only 0.5% of the region (Fig 5B), mostly in north of area. *T*_*min*_ showed a significant increasing trend (*p<*0.05) in northeast (Fig 5B).

The area that has an increasing trend of NDVI in winter from 1982 to 2015 was 72.7%. 48.3% of the area has a significant increasing trend (*p*<0.05). The areas showing non-significant increasing trends of NDVI in winter (*p*>0.05) were mainly distributed throughout the area (Fig 5C).

### 3.2. Partial correlation between the NDVI and seasonal asymmetric warming

#### 3.2.1. Region analysis

The partial correlation coefficient between the NDVI and the seasonal *T*_*max*_*/T*_*min*_ (Table 2) showed that the second-order partial correlation coefficient of the NDVI and *T*_*max*_ was positive for the area as a whole. Among them, the NDVI and *T*_*max*_ showed an extremely significant positive correlation in spring and winter (*p* < 0.01), a significant positive correlation in autumn (*p <* 0.05), and no correlation in summer (*p* > 0.05).

**Table 2:** Partial correlation coefficients between NDVI and *T*_*max*_ and *T*_*min*_ for different vegetation types in different season over the Loess Plateau.

Season	Spring		Summer		Autumn		Winter	
Region	***T***_***max***_	***T***_***min***_	***T***_***max***_	***T***_***min***_	***T***_***max***_	***T***_***min***_	***T***_***max***_	***T***_***min***_
	0.702 **	0.707 **	0.270 ^NS^	0.343 *	0.345 *	0.291 ^NS^	0.579 **	0.453 **
Cultivated Land	0.658 **	0.705 **	0.233 ^NS^	0.363 ^NS^	0.355 *	0.358 *	0.579 **	0.463 **
Forest	0.742 **	0.782 **	0.182 ^NS^	0.158 ^NS^	0.318 ^NS^	0.291 ^NS^	0.580 **	0.378 *
Grasslands	0.635 **	0.582 **	0.315 ^NS^	0.384 *	0.299 ^NS^	0.139 ^NS^	0.498 **	0.369 *
Shrublands	0.547 **	0.409 *	0.139 ^NS^	0.203 ^NS^	0.083 ^NS^	−0.061 ^NS^	0.277 ^NS^	0.242 ^NS^
Wetland	0.439 *	0.398 *	0.221 ^NS^	0.349 *	0.283 ^NS^	0.291 ^NS^	0.379 *	0.308 ^NS^

* *p <* 0.05,

** *p* < 0.01,

NS *p* > 0.05.

The second-order partial correlation coefficient of the NDVI and *T*_*min*_ passed the significance tests of *p* < 0.01 in spring and winter and *p* < 0.05 in summer and was non-significant correlated in autumn (*p* > 0.05). Thus, compared with the day-time temperature increase, the night-time temperature increases in summer had a more significant positive impact on the NDVI. In contrast, in autumn, the day-time temperature increase had a more significant impact on the NDVI than the night-time increase. *T*_*max*_ and *T*_*min*_ in spring had a more positive effect on the NDVI in the area.

The different types of NDVI showed partial positive correlations with *T*_*max*_ and *T*_*min*_ in spring (*p<*0.05) and non-significant partial positive correlations with *T*_*max*_ in summer (*p*> 0.05). In summer, grasslands and wetlands showed partial positive correlations with *T*_*min*_ (*p* < 0.05), while other types of NDVI showed non-significant partial positive correlations (*p*> 0.05). Except cultivated land, there was a non-significant partial positive correlation between the NDVI and *T*_*max*_ in autumn (*p* > 0.05). In autumn, cultivated land showed significant partial positive correlations with *T*_*min*_ (*p <*0.05), while other types of NDVI showed non-significant partial positive correlations (*p*>0.05). In winter, cultivated land, forest, and grassland showed partial positive correlations with *T*_*max*_ and *T*_*min*_ (*p<*0.05), shrub showed non-significant partial positive correlations with *T*_*max*_ and *T*_*min*_ (*p*>0.05), wetland showed a significant partial positive correlation with *T*_*max*_. In conclusion, different types of vegetation had significant differences in response to daytime and nighttime warming in different seasons.

#### 3.2.2. The pixel scale analysis

In order to understand the spatial pattern of the correlation between the seasonal NDVI and *T*_*max*_/*T*_*min*_ in the area, patterns for the correlation between seasonal NDVI and *T*_*max*_ or *T*_*min*_ were obtained.

In spring, the NDVI was positively correlated with *T*_*max*_ in 92.9% of the regions, of which 68.2% were significant at *p <* 0.05. Only 7.1% of the regions showed a negative correlation between the NDVI and *T*_*max*_, mainly in south and northwest however, these correlations were non-significant (*p* > 0.05). A positive correlation between the NDVI and *T*_*min*_ was shown in 90.6% of the area, with 58.0% being significant (*p <* 0.05). 9.4% of the area showed a negative correlation between the NDVI and *T*_*min*_ and did not pass the significance test (*p* > 0.05). The distribution range was the same as that described for negative correlations between the NDVI and *T*_*max*_. Thus, in general, daytime and nighttime warming in spring had positive effects on the growth of vegetation in most areas of the Loess Plateau, possibly due to a common advance of the vegetation growing season as a result of the rising temperature [30]. The area where the NDVI and *T*_*max*_ showed a positive correlation was slightly larger than that where the NDVI and *T*_*min*_ showed a positive correlation, possibly because a rising temperature in the day-time has a greater effect on the advancement of seedling and leaf development [31].

**Fig 6:**
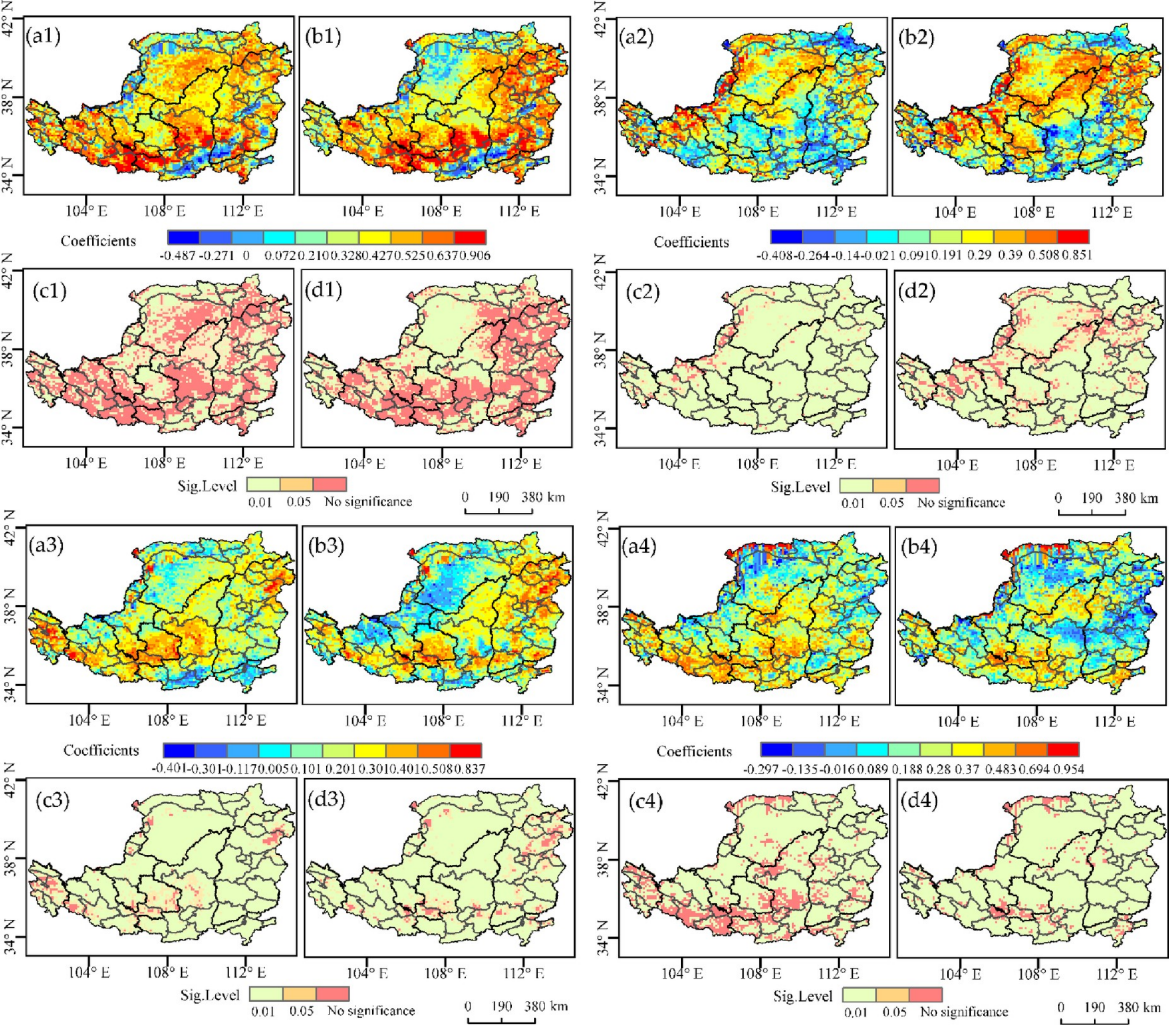
Spatial patterns of the correlations between NDVI and corresponding *T*_*max*_ or *T*_*min*_ over the Loess Plateau from 1982 to 2015 among seasons. (1) spatial patterns of the correlations between NDVI and corresponding *T*_*max*_ or *T*_*min*_ in spring; (2) spatial patterns of the correlations between NDVI and corresponding *T*_*max*_ or *T*_*min*_ in summer; (3) spatial patterns of the correlations between NDVI and corresponding *T*_*max*_ or *T*_*min*_ in autumn; (4) spatial patterns of the correlations between NDVI and corresponding *T*_*max*_ or *T*_*min*_ in winter;(a) The partial correlation coefficients between NDVI and *T*_*max*_. (b) The partial correlation coefficients between NDVI and *T*_*min*_;(c) significance level of the partial correlation coefficients between NDVI and *T*_*max;*_ and (d) significance level of the partial correlation coefficients between NDVI and *T*_*min*_.

The spatial distribution of the partial correlation coefficient between the NDVI and daytime and nighttime warming in summer (Fig 6) showed that 70.3% of the regions had a positive correlation between the NDVI and *T*_*max*._ 9.0% (*p <* 0.05) of the regions that passed the significance test were mainly distributed in the Northwestern Loess Plateau. 29.6% of the regions, mainly in the southeast of the area, showed non-significant, negative correlations between the NDVI and *T*_*max*_. 78.4% of the regions where the NDVI and *T*_*min*_ showed positive correlations and 25.8% of the regions that passed the significance test (*p* < 0.05) were distributed in the middle and northwest of the study area. 21.6% of the regions that showed a negative correlation between the NDVI and *T*_*min*,_ mainly in the south of this area, did not pass the significance test (*p* > 0.05). Thus, in general, a higher proportion of areas showed a positive impact on vegetation from daytime and nighttime warming as compared with those in which it had a negative impact. Compared with spring, the proportion of NDVI and *T*_*max*_ showing a significant positive correlation decreased significantly in summer, possibly because the day-time temperature in summer is close to the optimal temperature for vegetation growth in most areas, leading to lower sensitivity of the vegetation to day-time temperature increase in this season [32].

The spatial distribution of the partial correlation coefficient of autumn vegetation and daytime and nighttime warming (Fig 6) showed that 85.8% of the regions showed a positive correlation between the NDVI and *T*_*max*_, and the 12.6% (*p* < 0.05) that passed the significance test were mainly distributed in the west and south. 14.2% of the regions, mainly in the southwest of the study area, showed a non-significant negative correlation between the NDVI and *T*_*max*_. 75.8% of the regions, mainly in the east and south of the area, showed a positive correlation between the NDVI and *T*_*min*,_ with 13.6% passing the significance test (*p* < 0.05). 24.2% of the regions, mainly those in the northwest of the Loess Plateau, in Ordos, showed a non-significant negative correlation between the NDVI and *T*_*min*_. Thus, the temperature increases in autumn had a positive effect on vegetation in most areas. Compared with spring, the area showing a significant positive correlation between the NDVI and *T*_*max*_ decreased in autumn, possibly because the duration of vegetation photosynthesis and the sensitivity of vegetation productivity to temperature in autumn were lower [33].

The spatial distribution of the partial correlation coefficient of winter vegetation and daytime and nighttime warming (Fig 6) showed a positive correlation between the NDVI and *T*_*max*_ in 94.0% of the regions, and only 18.3% (*p <* 0.05) passed the significance test, mainly those in the southwest of the Loess Plateau (*p* < 0.05). 6.0% of the regions, mainly those in the northwest, showed a non-significant negative correlation between the NDVI and *T*_*max*_. The 80.9% of the regions where the NDVI and *T*_*min*_ showed a positive correlation, and the 4.7% of the regions that passed the significance test (*p <* 0.05) were mainly distributed in the south of the area. 19.1% of the regions, mainly those in the east of this area, showed a non-significant negative correlation between the NDVI and *T*_*min*_. Thus, daytime and nighttime warming in winter was conducive to vegetation growth in most areas, possibly because temperature, as a stress factor of vegetation growth in winter, is conducive to vegetation photosynthesis.

## 4. Discussions

In this study, daytime and nighttime warming is asymmetric among different seasons, which coincides with some previous studies [34]. This study found that seasonal NDVI in the Loess Plateau showed increasing trends, which is in agreement with by previous studies [35]. Fensholt et al [36] assessed the accuracy of the long term GIMMS3g NDVI data using MODIS NDVI for an overlapping period of 2000–2010 and concluded that the temporal trends derived from GIMMS NDVI are in overall good agreement with trends from MODIS NDVI data in most of the world's semi-arid, dry sub-humid, and sub-humid areas.

We found that daytime and nighttime warming over the Loess Plateau had a positive effect on NDVI during 1982–2015. Specifically, the response of the NDVI to daytime and nighttime warming was significantly different in different seasons. Among all the seasons, spring shows the largest area with a significantly positive relationship between *T*_*max*_ and the NDVI (Fig 6). The availability of soil nitrogen was improved in spring [37]. There are earlier and longer growing seasons driven by temperature, which can promote vegetation photosynthetic activity [30]. The area with a significantly positive relationship between *T*_*max*_ and the NDVI during winter was smaller than that in the other seasons, because of lower soil moisture and surface soil frost in some regions [38]. Winter does not include growing seasons (April-October). As such, the temperature sensitivities of the photosynthetic activity and productivity of winter are both smaller than that of the other three seasons. Summer shows a smaller area, with a significantly positive relationship between *T*_*max*_ and the NDVI (Fig 6), compared to spring and autumn. The analysis suggests that *T*_*max*_ of summer in most areas is close to the optimal value for plant photosynthetic activity [30]. Fensholt, et al [36] gave an explanation for the smaller response between the NDVI and *T*_*max*_ in summer than that in spring and autumn, i.e., it is associated with the saturation of the NDVI over dense vegetation in summer. Similar to *T*_*max*_, *T*_*min*_ may have impacted on vegetation photosynthetic activity in opposite ways, through the action of different mechanisms [39–40]. The study showed that the positive correlation between *T*_*min*_ and the NDVI is mainly evident in spring and summer, implying that the dominant ecophysiological effects of *T*_*min*_ vary seasonally.

In most regions, *T*_*max*_ and *T*_*min*_ had promoting effects on the NDVI, and in a small number of regions the day-night temperature rise had an inhibiting effect on the NDVI. Most vegetation photosynthesizes during the daytime [3]. In semi-humid areas with excess moisture and areas in which temperature is the main limiting factor to vegetation growth, an increase in *T*_*max*_ can promote the opening of stoma in vegetation leaves and vegetation transpiration, therefore also increasing the probability of CO_2_ and improving the availability of soil nitrogen. This promotes the growth of vegetation, characterized by an NDVI increase [41]. An increase in *T*_*max*_ in the arid areas of the Loess Plateau can accelerate leaf transpiration, leading to increased evaporation, reduced soil moisture, exposure to water stress, and inhibition of growth and photosynthesis. This is consistent with the research results of Peng et al. [3], who found that an increase of *T*_*max*_ was beneficial to vegetation growth and its ecosystem carbon sink function in most cold and humid regions, but not to the vegetation growth in temperate arid and semi-arid regions.

An increase in *T*_*min*_ at night can decrease the volume of endosperm cells in the mature stage of vegetation [42] and shorten the grain-filling stage of vegetation [43] by increasing the rate of vegetation autotrophic respiration [44]. This has a negative effect on the promotion of the NDVI in vegetation. The increase in *T*_*min*_ can improve vegetation resistance to drought [45] and regulate the content of carbohydrates in plant leaves [46] by reducing the frequency of frost disasters [35]. This has a positive impact on vegetation productivity.

Since the photosynthesis of most vegetation takes place during the day and the respiration of vegetation runs throughout the day, asymmetric warming will inevitably affect the carbon absorption and consumption of vegetation [47]. The various types of vegetation showed partial positive correlations with *T*_*max*_ and *T*_*min*_ in spring (*p <* 0.05), likely due to the advancement of seedling establishment and sprouting leaves. There was a non-significant partial correlation between various types of NDVI and *T*_*max*_ in summer. Except for cultivated land, there was a non-significant partial correlation between other types of vegetation and *T*_*max*_ in autumn. In most areas, the day-time temperature in summer and autumn was close to the optimum for vegetation growth, reducing the sensitivity of vegetation to day-time warming. Cultivated land, forest, and grassland all showed positive partial correlations with *T*_*max*_ and *T*_*min*_ in winter (*p <* 0.05), shrub showed a non-significant partial correlation with *T*_*max*_ and *T*_*min*_ in winter (*p* > 0.05), wetland showed a significant positive partial correlation with *T*_*max*_ in winter. Different types of vegetation had significant differences in daytime and nighttime warming in winter. In winter, the rising temperature was conducive to the photosynthesis of large vegetation. Meanwhile, the decrease in rainfall and water impaired the growth of vegetation.

The NDVI is a comprehensive response to climate change. This paper mainly discussed precipitation and temperature under hydrothermal climate conditions. Future research should comprehensively consider the impacts of humidity, evaporation, sunshine duration, and other meteorological factors on the NDVI. In recent years, ecological projects have been carried out in the Loess Plateau, such as returning grazing land to grassland, which will lead to changes in land cover and subsequent changes in the NDVI [48]. Determining how to quantitatively eliminate the dynamic changes in vegetation caused by land cover change is an important issue for further research. This paper used 8 km×8 km mixed pixel NDVI data, which comprehensively reflected the spectrum of all land cover. However, the next step is to combine other higher resolution image data, such as MODIS NDVI. Data-fusion that was carried out on images from 1982–2000 and high resolution remote sensing image data from 2000–2015 can be combined to obtain nearly 34 years of high resolution image datasets to allow further study on the responses of vegetation dynamics to seasonal daytime and night-time asymmetric warming. Meanwhile, such effects can be further quantified by combining the observation experimental data of ground stations and model simulation analyses to provide a more reliable scientific basis for ecological environment monitoring and adaptation to climate change.

## 5. Conclusion

In the Loess Plateau, the daytime and nighttime temperatures in each season showed significant (*p<* 0.05) increasing trends over the period 1982-2015except for *T*_*max*_ in autumn. The increasing speed of *T*_*max*_ and *T*_*min*_ were asymmetrical across Different seasons, and have not been evenly distributed in this area. For example, the increasing rates of *T*_*min*_ in spring, summer, autumn, and winter were 0.75, 1.20, 1.88, and 1.10 times larger than that of *T*_*max*_, respectively. Secondly, the NDVI in the Loess Plateau was extremely significant (*p*< 0.01) positively correlated with *T*_*max*_ and *T*_*min*_ in spring and winter, and with *T*_*min*_ in summer and *T*_*max*_ in autumn across entire Loess Plateau. Meanwhile, the different vegetation had different responses to seasonal daytime and nighttime asymmetric warming. Whether it increasing of the *T*_*max*_ or *T*_*min*_ in spring, it will promote the growth of vegetation. Only grasslands and wetland benefitted from increasing of *T*_*min*_ in summer. Autumnal increasing of *T*_*max*_ or *T*_*min*_ merely helped cultivated land growth. Winter increasing of *T*_*max*_ or *T*_*min*_ could promote growth of cultivated land, forest, and grassland, while only wetland showed a significantly positive partial correlation with *T*_*max*_ in winter.

The results suggested that vegetation variability to asymmetric warming is important to understand the changes in vegetation photosynthetic activity under global warming and ultimately influences regional and hemispheric-scale carbon balances. Given climate warming is projected to further increase in the future, our results, which addresses an improved characterization vegetation variability to asymmetric warming under the temporal and spatial scales, provide a valuable reference for assessing and predicting underlying mechanisms of vegetation variability to global climate change.
